# Coal Gasification Slag as a Green Additive in Supplementary Cementitious Materials: Mechanical Properties and Microstructure

**DOI:** 10.3390/ma18010086

**Published:** 2024-12-28

**Authors:** Hong Yang, Hailong Wang

**Affiliations:** 1 College of Water Conservancy and Civil Engineering, Inner Mongolia Agricultural University, Hohhot 010018, China; yanghong@emails.imau.edu.cn; 2 Autonomous Region Collaborative Innovation Center for Integrated Water Resources and Water Environment Management in Inner Mongolia Section of Yellow River Basin, 306 Zhaowuda Road, Hohhot 010018, China

**Keywords:** cement, coal gasification slag, supplementary cementitious materials, compressive strength, hydration products

## Abstract

Gasification slag is the solid waste produced in the process of coal gasification. China produces approximately 30 million tons of gasification slag every year, which urgently needs to be recycled in an efficient and sustainable way. This paper discusses the feasibility of using gasification slag as a supplementary cementitious material (SCM). The working properties, mechanical properties, and microstructure of cement paste after the addition of gasification slag were studied and compared with those of pure cement paste. The results indicate that the hydration products of the composite paste contain a significant amount of Ca(OH)_2_ and C-S-H gel when the content of gasification slag is less than 30%. However, when the gasification slag content exceeds 30%, the primary hydration product shifts to the C-A-S-H gel. Furthermore, the C-(A)-S-H gel tends to exhibit a lower calcium–silicon ratio and a higher degree of polymerization as the gasification slag content increases. Specifically, the Ca/Si ratio of the 60% C-A-S-H gel is 1.66, with a degree of polymerization of 0.77. When the gasification slag content is maintained at or below 30%, the compressive strength of the gasification slag cement paste decreases by approximately 3.7% to 9.3% compared with that of Portland cement (PC). Nevertheless, the composite cement meets the design requirements of 42.5 composite Portland cement. Thus, gasification slag has emerged as a promising supplementary cementitious material (SCM), with significant potential for widespread application.

## 1. Introduction

Cement-based materials have good mechanical properties, low economic costs, and the source of raw materials needed to meet the needs of large-scale industrial production, so concrete, cement mortar, and other cement-based materials in the world are the most widely used and most widely used building materials. However, the production process of cement materials involves high levels of pollution and high energy consumption, consuming many nonrenewable resources, such as limestone, clay, and coal, while also emitting high levels of CO_2_, SO_2_, other exhaust gases, and dust [1]. China is currently at the peak of infrastructure construction, and the demand for cement continues to increase. However, the energy crisis and environmental problems have become increasingly prominent, which has brought more urgent challenges and pressures in terms of resources and the environment to the development of the cement industry. Therefore, low-carbon, green, and circular development has become the principle and direction that the scientific development of the cement industry in the new era must follow [2]. The cement industry urgently needs to solve the problem of reducing the high energy consumption and emissions in the Portland cement production process. Therefore, the building materials industry urgently needs new green cementing materials with low energy consumption and low CO_2_ emissions.

Studies have shown that the partial replacement of cement with supplementary cementitious materials (SCMs) is considered one of the most effective and practical long-term solutions for reducing the environmental burden associated with cement production. This method can effectively reduce the energy consumption and carbon emissions of cement materials in the production process [3,4]. SCMs are mainly derived from industry, agriculture, and the ocean. A large amount of industrial and agricultural waste is stored or disposed of in landfills, but these wastes have potential resource utilization value. Research has shown that fly ash, silica fume, slag, and rice husk ash have all been successfully used in concrete to improve its working and mechanical properties while also reducing carbon dioxide emissions [5,6,7]. Chen et al. [8] discovered that using walnut shell ash (WSA) as a supplementary cementitious material can improve pozzolanic activity and setting time in cement paste when the content is less than 20%. Although SCMs help save energy and reduce CO_2_ emissions, their supply cannot meet the growing demand of the construction industry [9]. Therefore, it is necessary to explore new SCMs to enrich their types.

Coal combustion emits a large amount of greenhouse gases, which harm the environment, so it is critical to use coal cleanly and efficiently [10]. Coal gasification is one of the key technologies for utilizing coal efficiently and cleanly, but this process results in the generation of a large amount of solid waste residue and high-salt wastewater [11,12]. Coal gasification slag is a solid residue that emerges from a series of physical and chemical alterations of inorganic minerals in coal, along with carbon residue particles in coal, during the incomplete combustion of coal with oxygen or oxygen-rich air to generate CO and H_2_. It can be categorized into coarse slag and fine slag. Depending on the different coal gasification processes, the generation temperature of gasification slag ranges from 1400 to 1700 °C. China currently produces approximately 30 million tons of gasification slag per year, with the primary treatment methods being storage and landfill, which have yet to achieve large-scale comprehensive utilization, resulting in serious environmental pollution and waste of land resources [13]. The resource utilization of gasification slag at home and abroad is focused primarily on building materials, soil improvement, and the preparation of high-value materials, with the use of construction materials being an important way to consume gasification slag on a large scale [14]. The main chemical composition of the gasification slag is SiO_2_, Al_2_O_3_, CaO, Fe_2_O_3_, etc., which is similar to the composition of Portland cement. The silica–aluminum–calcium component of the gasification slag exists in the form of an amorphous glass phase, and its gelling activity can be stimulated under certain conditions [15]. Fu et al. [16] reported that when the content of coal gasification slag is less than 10%, it can play a role in nucleation and volcanic ash in cement paste. Compared with PC without CGS, the 1d, 7d, and 28d strength of G1P increased by 7.1%, 6.9%, and 5.4%, respectively. Unreacted gasification slag mainly exists in an agglomerated state. Luo et al. [17] investigated the effects of coal gasification slag, both coarse and fine, on cement paste hydration and microstructure. The strength activity index of CGA and CGS was 100.9% and 82.7%, respectively. Currently, there are few reports on coal gasification slag as an auxiliary cementing material, and it is difficult to use coal gasification slag as SCMs in large quantities without carbon ash separation [14].

This study investigated the performance and mechanism of cement paste using decarbonized gasification slag as a supplementary cementitious material, revealing the molecular structure and degree of polymerization changes of hydration products. First, the working performance of the cement paste with gasification slag was evaluated, and the effect of the gasification slag content on the hydration heat release of the cement paste was investigated via isothermal calorimetry. The mechanical properties of different cement pastes were measured in terms of compressive strength. The microstructures of various cement pastes were analyzed using X-ray diffraction (XRD), thermogravimetric (TG) analysis, mercury injection (MIP), Fourier transform infrared (FTIR) spectroscopy, and scanning electron microscopy (SEM).

## 2. Materials and Methods

### 2.1. Raw Materials

**Figure 1:**
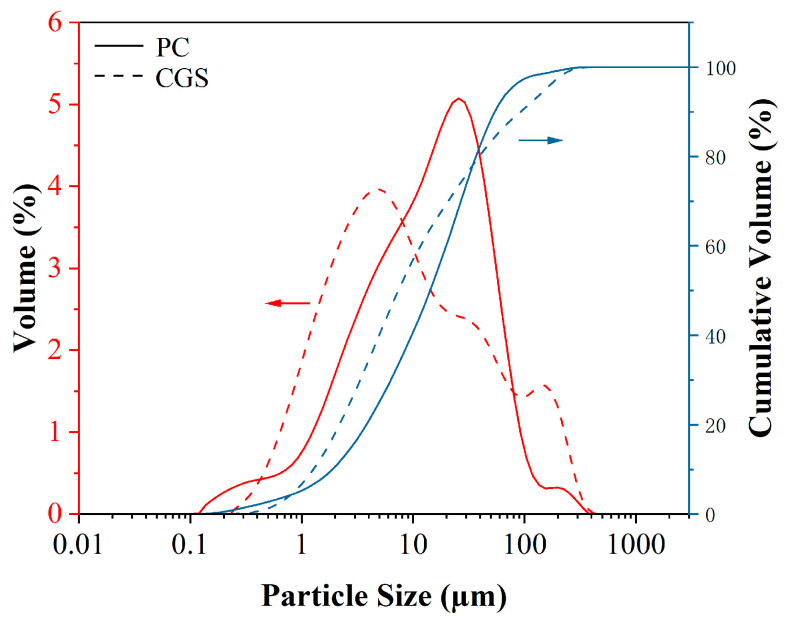
Particle size distributions of PC and CGS.

**Figure 2:**
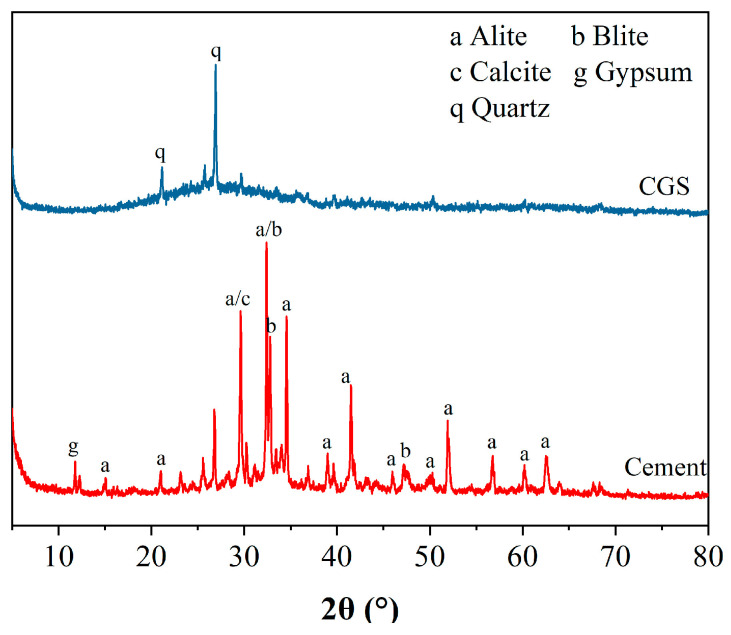
XRD patterns of different raw materials.

Coal gasification slag (CGS) is provided by Inner Mongolia National Energy Baotou Coal Chemical Co., Ltd. (Baotou, China). First, the gasification slag is burned for 2 h at 600 °C in the atmospheric environment to remove residual carbon. The cement (PC) was purchased from a commercial company and is 42.5 ordinary Portland cement of the Chinese national standard GB 175-2023 [18]. The chemical composition of the sample is analyzed using X-ray fluorescence spectroscopy (XRF) of the PANalytical Axios model (Almelo, The Netherlands). The chemical compositions of the coal gasification slag and cement were determined using X-ray fluorescence (XRF) spectrometry [19], as shown in Table 1. The particle size distributions of the two materials obtained via laser particle size analysis are shown in Figure 1. The D50 values of coal gasification slag and cement are 7.37 μm and 14.2 μm, respectively. An automatic specific surface and porosity analyzer (Micromeritics ASAP 2460, Norcross, GA, USA) was employed to test the specific surface area of the material. The adsorbed gas was N_2_, the degassing temperature was 300 °C, and the degassing time was 8 h. The specific surface areas of cement and coal gasification slag were 0.65 m^2^/g and 1.73 m^2^/g respectively. The phase composition of the cement and coal gasification slag was tested using a D8 Advance X-ray diffractometer (Bruker-AXS, Karlsruhe, Germany) with a scanning range of 2θ = 5–80° and a scanning speed of 2°/min. The XRD patterns of the cement and coal gasification slag are shown in Figure 2. All samples were in powder form and were sifted through a sieve with a pore size of 75 µm.

### 2.2. Specimen Preparation

The design of the test mix is detailed in Table 2. In this study, a water-to-binder (w/b) ratio of 0.4 was employed. The mass fractions of gasification slag were set at 15%, 30%, 45%, and 60%, with pure cement (100%) serving as the control group. Water was first added to the mixing pot, followed by cement and coal gasification slag, and the mixture was thoroughly blended. The resulting paste was then poured into 20 mm cubic steel molds and compacted on a vibrating table for 2 min. After 24 h of curing in a standard environment (20 ± 2 °C, RH ≥ 95%), the molds were removed. Subsequently, the samples were transferred to a curing chamber (20 ± 2 °C, RH ≥ 95%) until testing.

### 2.3. Test Methods

#### 2.3.1. Workability

A Vicat needle test was used to determine the setting time of the samples with a Vicat apparatus according to GB/T 1346–2019 [20]. Before testing, the paste was cast into a circular truncated cone-shaped Vicat mold with a height of 40 mm, a top inner diameter of 65 mm, and a bottom inner diameter of 75 mm.

The flow test of different cement pastes is based on the GB/T 8077-2012 [21] test. Fresh cement pastes were prepared according to Table 2. Fresh mixed cement paste was poured into a metal cube-shaped round mold with an upper diameter of 36 mm, a lower diameter of 60 mm, and a height of 60 mm; the mold was scraped flat with a scraper; the round mold was lifted in the vertical direction; the diameter of the paste was measured after 30 s; two perpendicular directions of the paste were selected; and the average value was taken as the result of fluidity.

#### 2.3.2. Heat of Hydration

The heat flow and cumulative heat release of different cement pastes at 20 °C were measured using the TAM Air eight-channel microcalorimeter of the TA Company (New Castle, DE, USA). After the cementified material was mixed with water according to Table 2, it was immediately placed into the isothermal calorimeter channel, and then the heat flow and cumulative heat release of the cement paste were continuously measured for 72 h.

#### 2.3.3. Compressive Strength

The compressive strength of the samples cured for 3, 7, and 28 days was tested using a YAW-300 pressure testing machine (Wuxi, China). The loading rate was 0.6 kN/s. Three parallel samples were selected from each group, and the average value was used as the compressive strength result. The internal small pieces of the broken sample were soaked in alcohol for 72 h to terminate hydration, and the alcohol was changed every 24 h. Then, the samples were removed and dried in a vacuum drying oven at 40 °C for 6 h, and relevant microscopic tests were carried out.

#### 2.3.4. Phase Changes and Microstructure

The phase composition of the cement paste was examined by means of a D8 Advance X-ray diffractometer (Bruker-AXS, Karlsruhe, Germany) featuring a Cu target (λ = 1.54 Å), an acceleration voltage of 40 KV, a tube current of 40 mA, a scanning speed of 2°/min, and a 2θ testing angle ranging from 5° to 80°. TG was carried out using the NETZSCH STA 2500 type thermogravimetric analyzer, produced by the German NETZSCH company (Bavaria, Germany), with a temperature testing range of 30–1000 °C, a heating rate of 10 °C/min, and a protective atmosphere of N_2_. The porosity of different cement pastes at 28 days was tested with Auto Pore IV 9500 (Norcross, GA, USA), and the samples were taken as small pieces approximately 5 mm in size. Fourier transform infrared spectroscopy (FTIR, Thermo Scientific Nicolet 6700, Waltham, Ma, USA) was employed to analyze the functional groups and elemental bonding of the reaction products. The test samples were mixed and ground with KBr of a mass fraction of 1.0–1.2% and then pressed into pellets for testing within the wavelength range of 4000 cm^−1^ to 400 cm^−1^ (spectral resolution 2 cm^−1^), with 32 scans. The micromorphology of the samples was observed using a field emission scanning electron microscope (ZEISS GeminiSEM 300, Oberkochen, Germany). The test samples must be kept dry and sputter-coated with gold before testing using a high-resolution sputter coater with Pt as the sputtering target material at a sputtering current of 20 mA and a duration of 150 s. The samples of XRD, TG, and FTIR were in powder form and were sifted through a sieve with a pore size of 75 µm.

## 3. Results and Discussion

### 3.1. Workability

**Figure 3:**
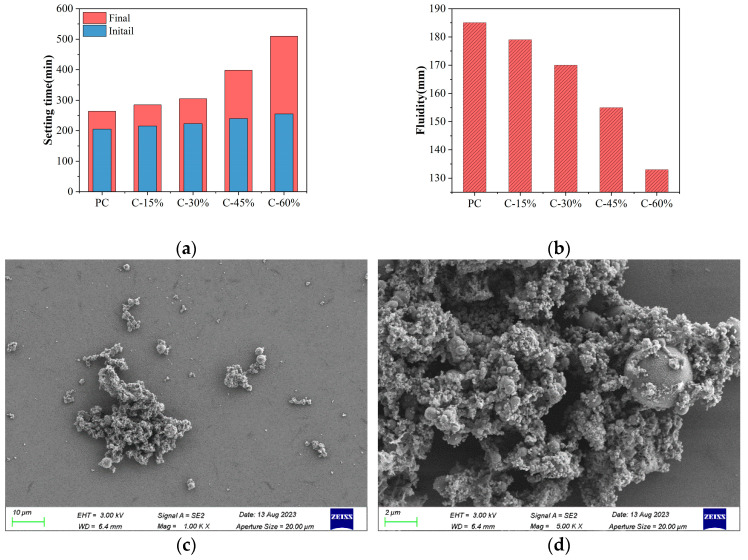
Workability of different cement pastes: (**a**) setting time; (**b**) fluidity; (**c**) SEM image of the gasification slag surface; (**d**) SEM amplification image.

The setting times of the different cement pastes are shown in Figure 3a. Notably, when the gasification slag content is less than 30%, its effect on the initial and final setting times of the cement paste is minimal. However, when the content exceeds 30%, there is considerable extension in both the initial and final setting times of the cement paste. Compared with those of PC, the initial and final setting times of C-60 were prolonged by 24.4% and 93.2%, respectively. An increase in the gasification slag content led to a decrease in the C_3_A content in the cement paste. Simultaneously, the lack of Ca(OH)_2_ required for hydration causes the gasification slag to have low pozzolanic activity during the initial stages of hydration. This adversely affects the reaction kinetics of the cement paste, leading to a prolonged setting time [16]. Similar to the changes in the setting time of other SCMs, such as silica fume [22], walnut shell ash [8], and sewage sludge ash [23], the setting time of paste is prolonged by replacing cement. In accordance with GB/175-2023 [18], ordinary Portland cement mixed with mineral admixtures must have an initial setting time of no less than 45 min and a final setting time of no more than 600 min. When the gasification slag content is less than 60%, the composite cement paste can meet the standard set for ordinary Portland cement in terms of both the initial and final setting times.

The fluidity results of the different cement pastes are shown in Figure 3b. As the gasification slag content increases, the fluidity of the cement paste gradually decreases. Specifically, with a gasification slag content of 60%, the fluidity of the cement paste is reduced by 28.1% compared with that of PC. This reduction aligns with the observed decrease in fluidity when cement paste is mixed with other waste residues, such as walnut shell ash [8] and rice shell ash [24]. The decrease in the fluidity of gasification slag cement paste is influenced by the particle size, surface properties, shape, and particle packing density of the gasification slag [25,26]. First, gasification slag has a smaller D 50 than cement (Figure 1). The BET specific surface area of the cement is approximately 0.65 m^2^/g, whereas that of the gasification slag is approximately 1.73 m^2^/g. The high specific surface area of the gasification slag allows it to absorb more water, resulting in a larger water film area [26]. As a result, the water content on the surface of the cement clinker decreases, leading to a reduction in the fluidity of the cement paste. On the other hand, the micro-morphology of the gasification slag particles exhibits an irregular bulk and porous structure characterized by the coexistence of carbon and ash. Embedded within this vitreous matrix are spherical glass particles composed primarily of calcium aluminosilicate, with minor amounts of iron and trace quantities of magnesium, potassium, and other minerals (Figure 3c,d). This unique surface characteristic enhances the viscosity of the gasification slag particles in the cement paste, thereby reducing its fluidity. To improve the fluidity of the paste in practical applications, the addition of a water-reducing agent can be employed.

### 3.2. Hydration Heat

**Figure 4:**
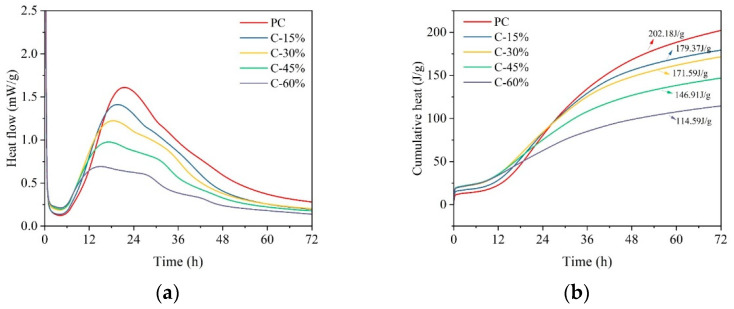
Hydration heat of different cement pastes: (**a**) heat flow and (**b**) cumulative heat.

The hydration heat results of the cement-based pastes with different gasification slag contents are shown in Figure 4. The ordinary cement (PC) group served as the control in this study, while the hydration characteristics of the gasification slag and cement mixture closely resemble those of traditional cement materials. The hydration process illustrated in Figure 4a can be categorized into four stages: dissolution, induction, acceleration, and deceleration, a classification commonly found in the literature [27]. Two distinct exothermic peaks can be observed in the hydration curves of all samples. The first exothermic peak appears within 3 h and is formed by the hydration of C_3_A. The second spike is due to the hydration of C_3_S. The acromion observed during the deceleration phase is associated with the conversion of AFt to AFm [28,29]. Notably, the main heat release peak of the paste containing gasification slag shifted to the left, and the occurrence time was earlier than that of the control group (PC). The maximum intensity of the main exothermic peak of C-60% is approximately 15 h, and the maximum intensity of the main exothermic peak of PC is approximately 21 h. This shift indicates the promotion of cement hydration by gasification slag, which is consistent with the findings of Fu et al. [16]. The increase in the rate of heat release when gasification slag is added to cement is attributed to the nucleation effect of the gasification slag, which provides more nucleation sites for cement hydration products. Studies have also shown that the impact of gasification slag on the hydration reaction of cement paste differs from that of other supplementary cementitious materials, such as limestone, steel slag, and granulated blast furnace slag [30,31,32,33,34]. It is important to highlight that the peak heat release of CGS paste occurs earlier compared to cement alone, while the setting time for CGS paste is extended. This phenomenon can be attributed to the fact that elevated temperatures accelerate chemical reactions and expedite water consumption within cementitious materials, thereby increasing the hydration rate. In the case of gasification slag paste, the accelerated hydration reaction generates more heat in the early stages, which may result in an earlier peak heat release. However, as heat is rapidly released, the subsequent hydration reaction may decelerate due to a temperature drop, consequently prolonging the setting time.

The cumulative heat release of cement-based pastes with different gasification slag contents is illustrated in Figure 4b. PC exhibited the highest total heat release at 202.18 J/g, whereas the total heat release of cement pastes containing gasification slag was lower, decreasing gradually with increasing gasification slag content. The total heat release of C-60% is 114.59 J/g, which is attributed to the dilution effect of the gasification slag. The cumulative heat release correlates with the changes in compressive strength, with similar values observed for C-15% and C-30%, explaining their comparable compressive strengths in the later stage.

### 3.3. Compressive Strength

**Figure 5:**
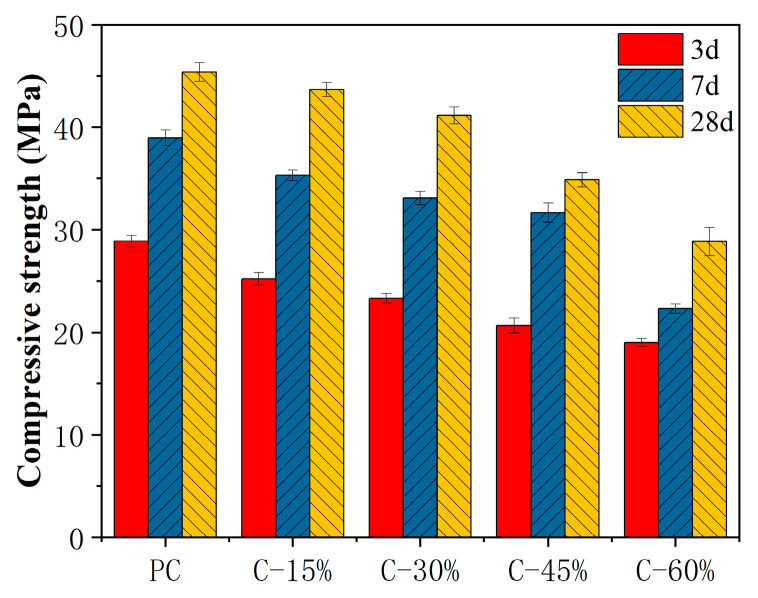
Compressive strength of different cement pastes.

The compressive strengths of different cement pastes at specific curing ages are shown in Figure 5. With increasing curing age, the compressive strength of cement pastes increases, whereas it decreases with increasing gasification slag content. In the phase where the gasification slag content ranges from 15% to 45%, the strength of the cement gasification slag slowly decreases. Compared with those of PC, the compressive strengths of C-15%, C-30%, and C-45% at 3 days decreased by 12.7%, 19.3%, and 28.4%, respectively, and those at 28 days decreased by 3.7%, 9.3%, and 23.2%, respectively. When the gasification slag content exceeds 50%, the strength decreases rapidly, with C-60% showing a decrease in compressive strength of 34.37% and 36.4% at 3 days and 28 days, respectively, compared with that of PC. These findings indicate that gasification slag has low pozzolanic activity in the early hydration stage. As the curing time increases, the amount of calcium hydroxide in the hydration products gradually increases, accelerating the pozzolanic reaction of the gasification slag. When the gasification slag content exceeds 50%, the dilution effect of the gasification slag on the cement is significant, leading to a reduction in the concentration of Ca^2+^ in the paste. This results in a significant decrease in the compressive strength of the paste due to the reduced amount of C-S-H gel and calcium hydroxide in the reaction products. These results are consistent with relevant research [16,17].

### 3.4. XRD

**Figure 6:**
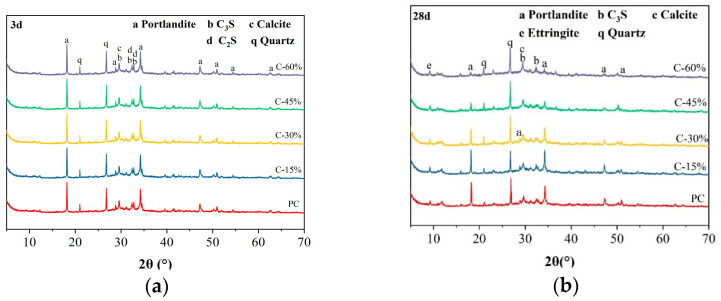
XRD patterns of different cement pastes at (**a**) 3 days and (**b**) 28 days.

The XRD patterns of different cement pastes at a specific curing age are shown in Figure 6. After 3 days of curing, the main hydration products of ordinary Portland cement paste (PC) include C-S-H gel, Ca(OH)_2_, and unhydrated cement clinker. The presence of calcite is related to carbonation occurring when CO_2_ in the air comes into contact with the hydration products. The addition of gasification slag had no significant effect on the crystal phases of the cement hydration products, and no new hydration products were found. The absence of ettringite on the 3 d XRD pattern may be due to insufficient hydration time and insufficient formation of ettringite to produce obvious diffraction peaks on the XRD pattern. After 28 days of curing, the main hydration products in the cement paste increased to include ettringite. The diffraction peak strength of Ca(OH)_2_ in gasified slag cement paste (C) is lower than that in pure cement paste (PC). With increasing gasification slag amount, this strength decreases. Two reasons may account for this change: First, Ca(OH)_2_ is a hydration product of cement, and decreasing the cement content results in a reduction in Ca(OH)_2_ generation. Second, the active SiO_2_ and Al_2_O_3_ in gasification slag react with Ca(OH)_2_ in volcanic ash, depleting Ca(OH)_2_ [16,17,35].

### 3.5. TG

**Figure 7:**
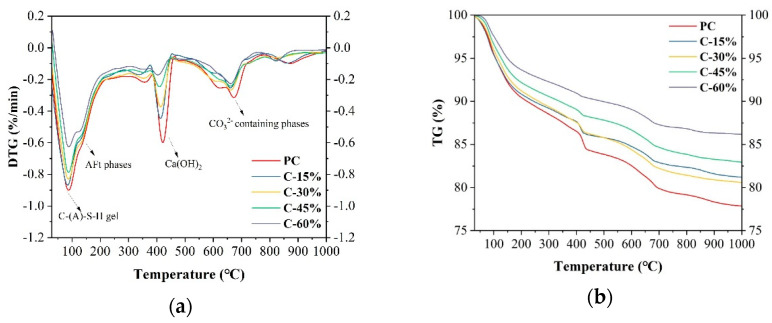
TG/DTG curves of different cement pastes at 28 days. (**a**) DTG curves; (**b**) TG curves.

The TG–DTG curves of different cement pastes at 28 days are depicted in Figure 7. In the DTG curve, all the cement pastes exhibited three distinct weight loss peaks. The first peak, in the range of 50–250 °C, primarily resulted from the dehydration of AFm, C-S-H gel, and Aft [36]. Relative to that of PC, the first peak strength of cement pastes containing gasification slag is lower, as evidenced by the lower mass loss in the TG curve. These results indicate that the addition of gasification slag hindered the formation of the C-S-H gel and diminished the strength of the sample. Since the pozzolanic activity of the gasification slag is low and cement serves as the primary cementing material, providing calcium, silicon, aluminum, and other elements for the generation of hydration products, the increased replacement of cement in the cement paste by gasification slag inevitably has a negative effect on the formation of hydration products. The second peak, occurring at 360–470 °C, is attributed to the dehydroxylation of calcium hydroxide. The peak strength of PC is the highest, and it decreases with increasing gasification slag content, which is consistent with the XRD results. The third peak, at approximately 700 °C, corresponds to the decomposition of the carbonate phase [37]. In general, the mass loss of the first and second peaks is related to the content of hydration products, and the compressive strength of the sample is related to the area of these peaks [38]. The specific results are shown in Table 3. The mass losses of the first and second peaks of the sample are 13.08%, 11.68%, 11.34%, 10.09%, and 8.33%, respectively, indicating a reduction in the generation of hydration products in the cement paste due to the addition of gasification slag. The similar mass losses of C-15% and C-30% explain the similar 28-day compressive strengths of the pastes. The second peak represents the decomposition of CH, with mass losses of 3.22%, 2.39%, 2.3%, 1.69%, and 1.32%, respectively. The mass loss rate decreases, indicating that the amount of Ca(OH)_2_ in the reaction product gradually decreases with the addition of gasification slag. This is due to the reduction in the cement content and the consumption of gasification slag.

### 3.6. MIP

**Figure 8:**
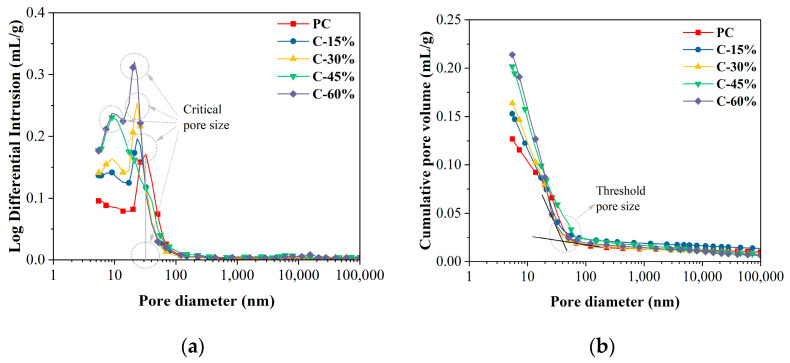
MIP results of different cement pastes at 28 days. (**a**) Pore size distribution; (**b**) cumulative pore volume.

The pore size distributions and cumulative pore volumes of the different cement pastes at 28 days are shown in Figure 8. Compared with those in the PC group, the number of holes < 20 nm (harmless holes) increased significantly after the addition of gasification slag, and the number of harmless holes increased with increasing gasification slag content. In addition, the threshold and critical pore size are calculated. The threshold pore size is the minimum continuous pore size of the sample obtained from the cumulative volume curve [39]. The critical pore size is the aperture corresponding to the peak height obtained from the aperture distribution curve [39]. The critical pore sizes of PC, C-15%, C-30%, C-45%, and C-60% were 32.31 nm, 23.07 nm, 23.89 nm, 9.24 nm, and 21.23 nm, respectively. After adding gasification slag, the critical pore diameter of the cement paste is reduced. Similarly, the threshold apertures of PC, C-15%, C-30%, C-45%, and C-60% were 50.22 nm, 38.32 nm, 36.84 nm, 66.5 nm, and 43.24 nm, respectively. The addition of gasification slag also significantly reduces the threshold pore size. This reduction in the critical pore size and threshold pore size indicates that the gasification slag fills and improves the pore structure of the cement paste and reduces the pore connectivity [40]. However, the cumulative pore volume of cement paste containing gasification slag is significantly greater than that of pure cement paste (PC).

**Figure 9:**
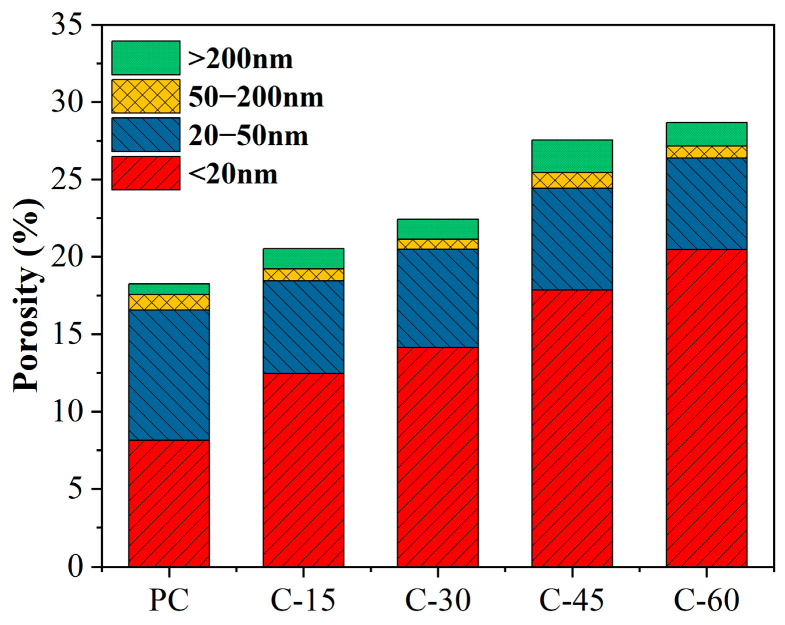
Porosity of different cement pastes at 28 days.

The utilization of supplementary cementitious materials alters the hydration process of cement-based materials and influences the development of the internal microstructure of the paste. The impact of this change depends on the dissolution rate and the quantity of supplementary cementitious material used [41]. According to the classification method of “harmless pores d < 20 nm, less harmful pores 20 < d < 50 nm, harmful pores 50 < d < 200 nm, and more harmful pores d > 200 nm” [42], the porosity results are illustrated in Figure 9. Gasification slag significantly impacts the total porosity and pore structure of cement paste. The total porosity of cement paste containing gasification slag exceeds that of pure cement paste (PC). When the gasification slag content is less than 15%, the pore structure of the cement paste can be improved, leading to a notable increase in the content of harmless and less harmful pores. This is the reason why the compressive strength of the C-15% group is comparable to that of the PC group. When the content of gasification slag exceeds 15%, porosity increases significantly. Compared with that of PC, the porosity of C-45% increased by 65.09%. With increasing content of gasification slag, the hydration products of cement decrease, the porosity of the paste increases, and the content of more harmful pores increases, which is the reason for the decrease in the compressive strength of the gasification slag cement paste. In ordinary cement paste, harmless pores (d < 20 nm) are categorized as gel pores, which are associated with the amorphous gel in the paste, leading to a densification of the paste structure and an increase in compressive strength. Notably, the content of harmless pores in the cement paste containing gasification slag is greater than that in the PC group. However, the compressive strength is lower in the former, possibly because the gasification slag is a porous material with a well-developed medium and large pores, which readily store water. During the preparation of fresh gasification slag cement paste, the pores of the gasification slag store water, and in the later curing process, the evaporation of water creates pores with d < 20 nm.

### 3.7. FTIR

**Figure 10:**
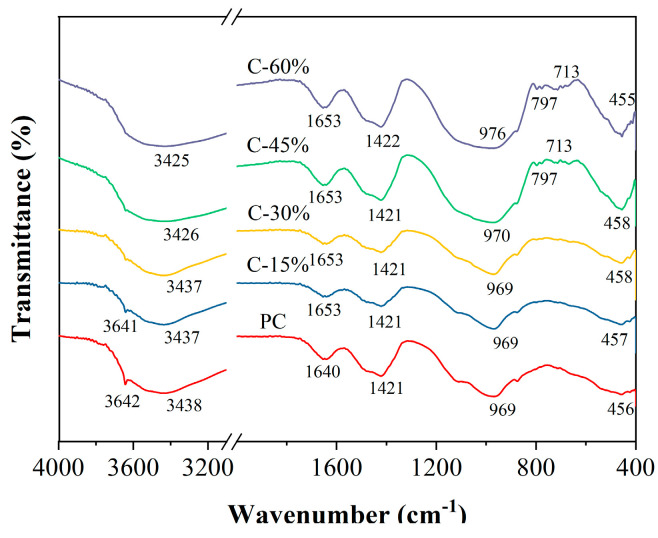
FTIR spectra of different cement pastes at 28 days.

The FTIR spectra of different cement pastes at 28 d are shown in Figure 10. The FTIR spectra of different pastes have similar vibration peaks, indicating that the gasified slag-cement paste and PC contain the same functional groups and bonding state. The absorption peak near 450 cm^−1^ corresponds to the internal bending vibration of the Si-O-Si bond, and the absorption peak near 713 cm^−1^ represents the internal stretching vibration of the T-O tetrahedron, with T representing Si or Al [43]. This is usually produced by the introduction of SiO_2_ into raw materials. The intensity of the absorption peak at approximately 450 cm^−1^ increases as the gasification slag content increases, suggesting an increase in the amount of unreacted gasification slag [44]. Conversely, the absorption peak near 713 cm^−1^ shows no significant change with increasing gasification slag content, indicating that the slag does not affect the bending vibration of the bond. The flexural vibration of the 4-coordinate AlO_4_ led to a change in the peak strength near 797 cm^−1^ [43]. Furthermore, the absorption peak near 970 cm^−1^ primarily results from the asymmetric stretching vibration of Si-O-T (Si or Al) in SiO_4_ [45], which is the characteristic peak of the C-S-H gel. The increase in slag content caused the absorption peak near 970 cm^−1^ to shift to a higher wavenumber, from 969 cm^−1^ for PC to 976 cm^−1^ for C-60%, which was primarily due to the change in the composition of the C-S-H gel. The relative molecular masses of Si and Al are similar, but the valence bond force constant of Al–O (0.75 angstroms) is smaller than that of Si–O (1.61 angstroms). As a result, the increase in the Si/Al ratio of the reaction products causes the asymmetric stretching vibration peak of Si-O-Si(Al) to shift towards a higher frequency [46]. Moreover, during this stage, the reaction process is rapid, leading to an increase in the number of reaction products and gel cohesion, which in turn results in an increase in the absorption frequency. The absorption peak near 1420 cm^−1^ corresponds to the in-plane and out-of-plane flexural vibrations of the O-C-O bond (CO_3_^2−^) [47], indicating carbonation during the storage and drying of all the samples. The peak at 1640 cm^−1^ represents the bending vibration of the H-O-H bond, which is associated with the chemically bound water in the hydration products [47]. Furthermore, the 3438 cm^−1^ peak is linked to the stretching vibration of -OH [48], whereas the 3644 cm^−1^ peak corresponds to the stretching vibration mode of Ca(OH)_2_ [48]. As the gasification slag content clearly increases, the intensity of the peak gradually decreases, and when the content reaches 60%, the peak almost disappears. This indicates that the Ca(OH)_2_ produced by the cement hydration reaction in the composite gel material was completely consumed by the gasification slag, which is consistent with the XRD analysis results.

**Figure 11:**
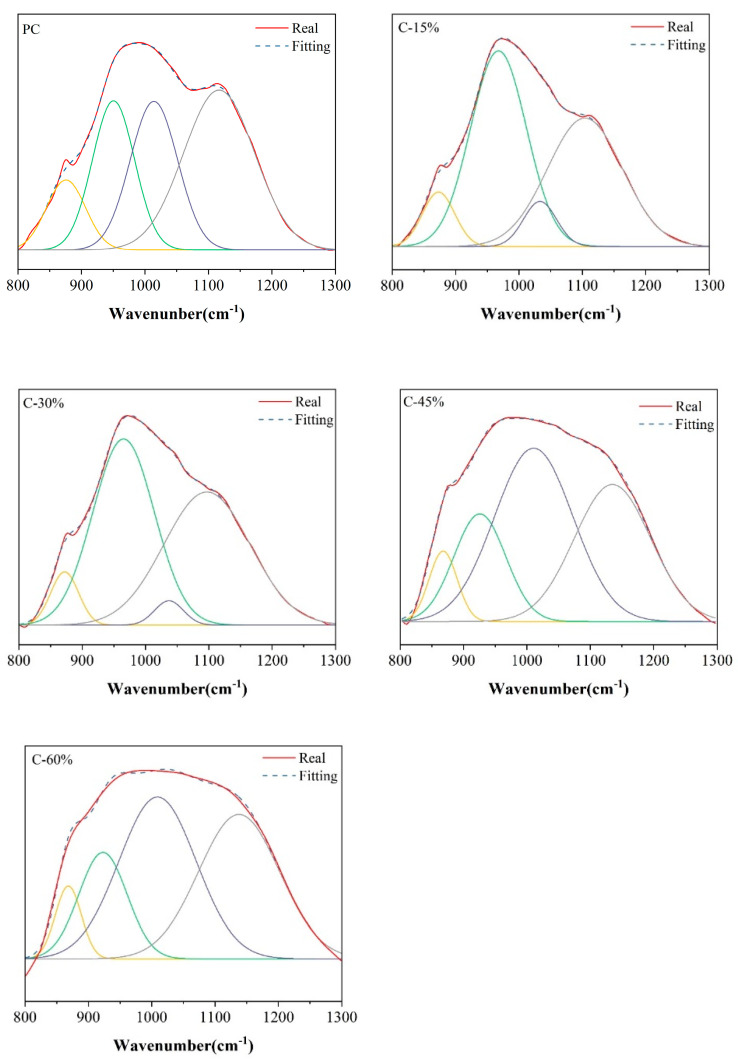
Peak fitting results for different cement pastes in the 800–1300 cm^−1^ range.

The FTIR spectrum provides insight into the vibration types of Si-O (Q^1^, Q^2^, Q^3^, and Q^4^) in C-S-H gels through band changes from 800–1300 cm^−1^ [49,50]. These vibration types are represented by the typical bands located at 850 cm^−1^, 970 cm^−1^, 1089 cm^−1^, and 1135 cm^−1^ in the spectrum [51,52]. The wavenumber and area changes of these characteristic peaks can be used to characterize the formation and transformation of hydrated silicate gels. The FTIR spectral bands in the range of 800–1300 cm^−1^ were fitted by Peak Fit v4.12 software, the peak area was calculated, and the polymerization degree (RBO) of different cement pastes was calculated according to Formula (1) [52]. The peak fitting results are shown in Figure 11, and the relevant parameters and RBO results of peak fitting are shown in Table 4. With increasing gasification slag content, the RBO of the C-S-H and C-A-S-H gels in the cement paste also increased. When the content of gasification slag is less than 30%, the RBO of the sample is lower than that of ordinary Portland cement (PC); when the content of gasification slag exceeds 30%, the RBO of the sample is greater than that of PC. Notably, there is an inconsistency between the degree of polymerization of a sample and its compressive strength. This may be due to the unique surface structure of the gasification slag, which leads to greater porosity of the paste.

(1) $$RBO=\left(1\times \frac{Q^{1}}{\sum Q^{n}}+2\times \frac{Q^{2}}{\sum Q^{n}}+3\times \frac{Q^{3}}{\sum Q^{n}}+4\times \frac{Q^{4}}{\sum Q^{n}}\right)=\frac{1}{4}\times \frac{\sum n\times Q^{n}}{\sum Q^{n}}$$

### 3.8. SEM and EDX

**Figure 12:**
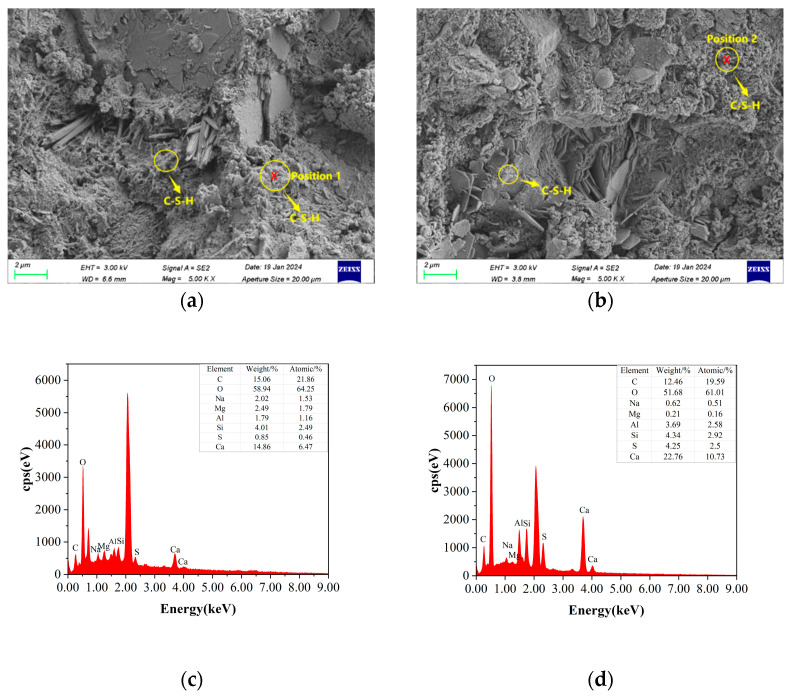

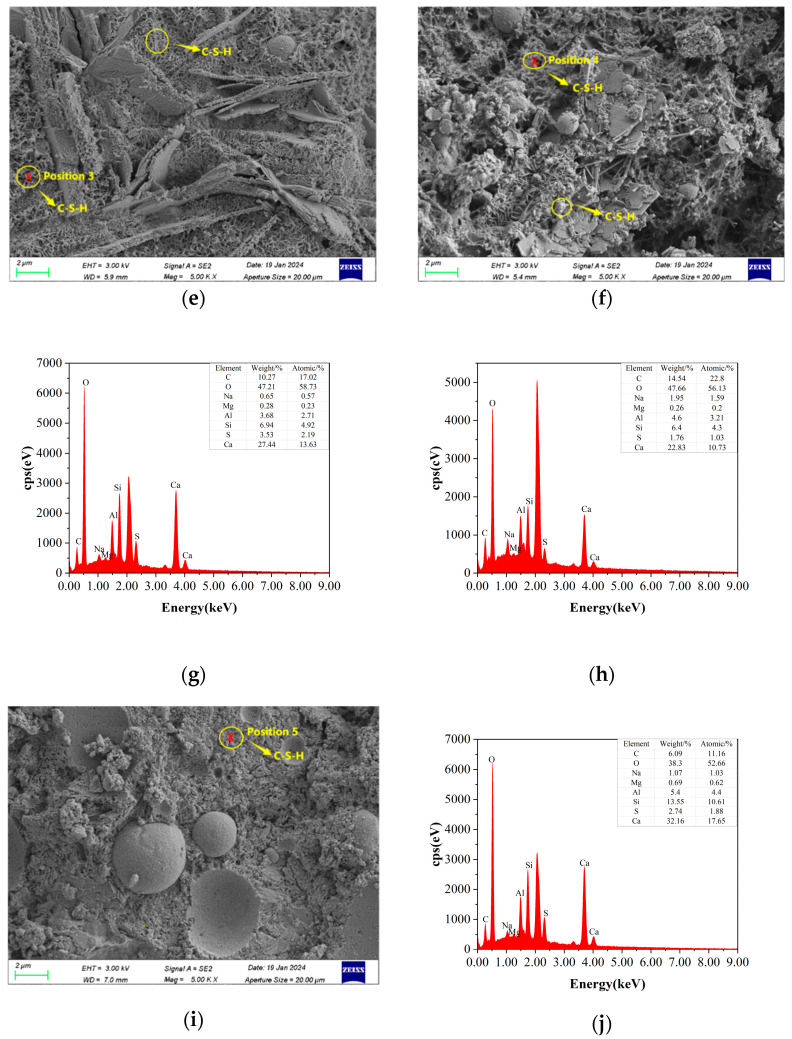
SEM images and EDX images of different cement pastes at 28 days. (**a**) PC; (**b**) C-15%; (**c**) EDX result at Position 1; (**d**) EDX result at Position 2; (**e**) C-30%; (**f**) C-45%; (**g**) EDX result at Position 3; (**h**) EDX result at Position 4; (**i**) C-60%; (**j**) EDX result at Position 5.

The SEM images and EDX images of different cement pastes at 28 d are shown in Figure 12. The hydration degree of the PC paste is relatively high, and hexagonal lamellar Ca(OH)_2_ [53], needle-like ettringite [54], and flocculent C-S-H gel [55] can be observed. Ettringite and C-S-H gel overlap and interlace with each other, forming a dense network structure with fewer pores. When the gasification slag content is less than 30%, it has a minor effect on the microstructure of the cement paste, leading to a relatively dense microstructure. However, when the gasification slag content exceeds 30%, the microstructure of the paste is significantly affected. Unreacted gasification slag is clearly present, along with a loose surrounding structure and numerous pores. As the slag content increased, the C-S-H gel and Ca(OH)_2_ decreased, the number of pores increased, and the structure became looser. These findings are consistent with the results of the MIP test. In C-45% and C-60%, some unhydrated gasification slag particles are deposited in the cement paste, causing the formation of more pores around these particles. This is the reason for the decrease in the compressive strength of the gasification slag cement paste.

According to the EDX data in Figure 12c, the main components of the PC paste are Ca, Si, O, and C-S-H gel, and the Ca/Si ratio is approximately 2.6. The main components of the colloidal gel of the cement paste containing gasification slag are Ca, Si, O, and Al, indicating that the gel product of the cement paste containing gasification slag is a C-(A)-S-H gel. The Ca/Si ratios of C-15%, C-30%, C-45%, and C-60% are 3.67, 2.77, 2.5, and 1.66, respectively. Mohan [56] proposed that when the Ca/Si ratio of the hydration product is less than 2.5, the hydration product is mainly a C-S-H gel. In contrast, the contents of Ca(OH)2, ettringite, and AFm in the hydration products increased. Compared with that of ordinary cement (PC), the Ca/Si ratios of C-15% and C-30% cement paste are greater, both greater than 2.5, which indicates that the hydration products of gasification slag cement paste contain more Ca(OH)2 and ettringite. When the content of gasification slag exceeds 30%, the main hydration product of the cement paste is the C-(A)-S-H gel. These findings indicate that the addition of gasification slag affects the structure of the C-S-H gel in the cement paste. The increase and decrease in the Ca/Si value are related to the degree of polymerization of the C-S-H gel, and the lower the ratio, the higher the degree of polymerization, which is consistent with the RBO results of FTIR.

## 4. Conclusions

In this work, the effects of the amount of gasification slag added on the working performance, heat of hydration, mechanical properties, and microstructure of cement paste are studied. The following conclusions can be drawn:

(1) The surface of the gasification slag is rough and porous, and it has low pozzolanic activity in the early stages of hydration. Therefore, with increasing content of the gasification slag, the setting time of the cement paste is extended, and the fluidity is reduced. When the content of gasification slag does not exceed 60%, the setting time of the paste can meet the requirements.

(2) The cement paste incorporating gasification slag initially enters an acceleration phase, during which the rate of heat release is maximized and the total accumulated heat release reaches its peak. However, both the peak value of the secondary heat flow and the cumulative heat release diminish as the content of gasification slag increases.

(3) When the content of gasification slag is less than 30%, the hydration products of the composite slurry predominantly consist of Ca(OH)_2_ and C-S-H gel. Conversely, when the gasification slag content exceeds 30%, the primary hydration product shifts to the C-A-S-H gel. As the proportion of gasification slag increases, the C-(A)-S-H gel transitions toward a lower calcium–silicon ratio and a higher degree of polymerization. The Ca/Si ratio for the C-60% C-A-S-H gel was measured at 1.66, with a polymerization degree (RBO) of 0.77.

(4) Due to the high specific surface area of the gasification slag, it easily absorbs water and forms a large water film, which leads to an increase in the porosity of the cement paste containing gasification slag. The density of the microstructure is reduced. Therefore, the compressive strength of cement paste containing gasification slag is reduced.

(5) Gasification slag has a detrimental effect on the hydration and mechanical properties of cement paste; however, when its content is maintained at or below 30%, the compressive strength of the gasification slag cement slurry is diminished by approximately 3.7% to 9.3% compared with that of ordinary Portland cement (PC). Nonetheless, this composite cement can still fulfill the design specifications for 42.5-grade composite Portland cement.

## Figures and Tables

**Table 1:** Chemical oxide compositions of the CGSs and PCs (%).

	CaO	SiO_2_	Al_2_O_3_	MgO	Fe_2_O_3_	SO_3_	Na_2_O	TiO_2_	K_2_O	Other	LOI
CGS	12.47	46.19	20.73	1.41	9.53	1.74	3.72	1.41	2.06	0.74	1.05
PC	54.96	22.82	8.04	3.56	3.86	4.62	0.32	0.53	0.77	0.52	1.96

**Table 2:** Mix proportions.

Paste ID	Cement	CGS	w/b
PC	100%	0%	0.4
C-15%	85%	15%	0.4
C-30%	70%	30%	0.4
C-45%	55%	45%	0.4
C-60%	40%	60%	0.4

**Table 3:** Mass loss of the first and second peaks.

Sample	Total Mass Loss (%)	The First Peak (%)	The Second Peak (%)
PC	13.08	9.86	3.22
C-15%	11.68	9.29	2.39
C-30%	11.34	9.04	2.3
C-45%	10.09	8.4	1.69
C-60%	8.33	7.01	1.32

**Table 4:** Peak fitting results.

Paste ID	Relative Content/%				RBO	R^2^
	Q^1^	Q^2^	Q^3^	Q^4^		
PC	9.952	22.912	25.681	41.455	0.746598	0.99
C-15%	7.378	45.906	6.25	40.467	0.69952	0.99
C-30%	5.954	45.63	2.8	45.636	0.720395	0.99
C-45%	6.24	17.635	42.968	33.156	0.757595	0.99
C-60%	6.062	16.485	40.031	37.419	0.772003	0.99

## Data Availability

The data presented in this study are available on request from the corresponding author. The data are not publicly available because they were tested in the laboratory.
